# Hemorrhage in Robot-assisted Partial Nephrectomy: A Multicentric, Retrospective Analysis of Blood Transfusion and Bleeding Requiring Intervention in 3726 Cases

**DOI:** 10.1016/j.euros.2026.04.010

**Published:** 2026-05-05

**Authors:** Olga Katzendorn, Hamza Idais, Mohammed R. Kanaan, Pouriya Faraj Tabrizi, Frank Schiefelbein, Georg Schoen, Clemens Wiesinger, Burkhard Ubrig, Simon Gloger, Philipp Nuhn, Ahmed Eraky, Christian Wagner, Abdirahman Ayanle, Claudia Kesch, Mulham Al-Nader, Boris A. Hadaschik, Johannes Friedemann Münden, Christian Fuhrmann, Markus A. Kuczyk, Stefan Siemer, Philip Zeuschner, Roman Mayr, Nina N. Harke

**Affiliations:** a Department of Urology, Hannover Medical School, Hannover, Germany; b Department of Urology, Klinikum Wuerzburg Mitte - Missioklinik, Wuerzburg, Germany; c Department of Urology, Urologische Klinik Muenchen - Planegg, Planegg, Germany; d Department of Urology, Klinikum Wels-Grieskirchen, Wels, Austria; e Department of Urology, Augusta-Kranken-Anstalt Bochum, Bochum, Germany; f Chair of Urology, University Witten/Herdecke, Bochum, Germany; g Department of Urology, University of Schleswig-Holstein Campus Kiel, Kiel, Germany; h Department of Urology, Icahn School of Medicine at Mount Sinai, New York, NY, USA; i Department of Urology, St. Antonius Hospital Gronau, Gronau, Germany; j Department of Urology, University Hospital Essen, Essen, Germany; k Department of Urology and Pediatric Urology, Saarland University, Homburg/Saar, Germany; l St. Josef Medical Center, University Medical Center Regensburg, Regensburg, Germany

**Keywords:** Hemorrhage, Bleeding, Complications, RAPN, Partial nephrectomy

## Abstract

**Background and objective:**

Postoperative bleeding in robot-assisted partial nephrectomy (RAPN) can result in a major complication. This study aims to identify factors associated with postoperative bleeding requiring an intervention (PBRI) as a major complication and predictors of blood transfusions as a minor complication.

**Methods:**

This study is a multicentric, retrospective analysis of 3726 RAPN cases. Separate comparative analysis was performed between: (1) patients with PBRI versus those without PBRI and (2) patients with blood transfusion versus those without blood transfusion. Two independent multivariable logistic regression analyses were performed to identify predictors for each endpoint.

**Key findings and limitations:**

PBRI occurred in 1.9% of patients and transfusion in 4.6%. Main interventions were angiography (49%) and operative revision (39%), with nephrectomy in 7.1% of patients. In both complication groups, patients were older (both *p* < 0.001), American Society of Anesthesiologists (ASA) scores were higher (both *p* < 0.001), and preoperative hemoglobin (Hb) was lower (both *p* < 0.05). Tumors in the transfused cohort were larger (*p* = 0.005), more frequently multiple (*p* = 0.01), and exophytic (*p* = 0.03). Patients with PBRI showed no difference in tumor characteristics. For both transfusion and PBRI, ischemia and operative time were longer (both *p* < 0.001) and surgeon’s expertise was lower (both *p* < 0.05).

Multivariable analysis showed a significant association of ASA score 3–4 (odds ratio [OR] 1.92, 95% confidence interval [CI] 1.29–2.88, *p* = 0.001) and longer operative time (OR 1.07, 95% CI 1.04–1.11, *p* < 0.001) with transfusion, and longer ischemia time (OR 1.04, 95% CI 1.01–1.07, *p* = 0.01) with PBRI. Patient’s age (*p* = 0.009 for PBRI; OR 1.03, 95% CI 1.01–1.04, *p* = 0.01 for transfusion) and lower preoperative Hb (OR 0.77, 95% CI 0.66–0.90, *p* = 0.001 for PBRI and OR 0.61, 95% CI 0.54–0.68, *p* < 0.001 for transfusion) were significantly associated with both complications.

Main limitation of this study is its retrospective design.

**Conclusion and clinical implications:**

Most hemorrhagic complications after RAPN can be managed with kidney-preserving, multidisciplinary approaches. However, whether preoperative anemia correction is associated with improved outcomes should be investigated in future studies.

## Take Home Message

Bleeding complications after robot-assisted partial nephrectomy are rare. Main factors associated with these complications are patient and intraoperative characteristics. Whether preoperative optimization of patient-associated risk factors, such as anemia, improves patient outcomes should be investigated in future studies.

ADVANCING PRACTICE **What does this study add?**

This study identifies clinical variables associated with bleeding complications after robot-assisted partial nephrectomy in a huge, multicenter cohort, and confirms that established interdisciplinary treatment strategies can preserve the operated kidney in most of the cases. Whether preoperative correction of some of these factors, such as preoperative anemia, reduces complication rates should be evaluated in further prospective studies.

**Clinical Relevance**

This large multicentric analysis demonstrates that hemorrhagic complications after robot-assisted partial nephrectomy are relatively uncommon (1.9% PBRI, 4.6% transfusion) and are predominantly manageable with kidney-preserving, minimally invasive strategies. Key patient-related (age, preoperative anemia, comorbidity) and intraoperative factors (operative and ischemia time) can help identify patients at higher risk, supporting improved perioperative planning and risk stratification.These findings highlight the potential role of preoperative optimization—particularly anemia correction—and surgical efficiency in reducing bleeding-related morbidity after RAPN, although prospective validation is needed. Associate Editor: M. Carmen Mir.

**Patient Summary**

In this study, higher American Society of Anesthesiologists score and longer operative time was associated with blood transfusion, longer ischemia time with bleeding requiring intervention, and patient age and low preoperative hemoglobin with both outcomes.

## 1 Introduction

Partial nephrectomy is the standard therapy for localized kidney cancer [1]. Robot-assisted partial nephrectomy (RAPN) is becoming increasingly common and represents a well-established, safe alternative to open or laparoscopic surgery [2]. However, complication rates up to 30%, with high-grade complications in 10%, have been reported previously [3].

Postoperative hemorrhage is a recognized complication after partial nephrectomy. Depending on the severity, management strategies involve conservative, interventional, or surgical approaches [4], [5]. Clinically severe bleeding requiring urgent interventional or surgical treatment occurs in approximately 2% of RAPN cases and represents a major, potentially life-threatening complication [6], [7]. Usual clinical manifestations include flank pain and/or hematuria. The underlying reasons are iatrogenic vascular injuries, such as pseudoaneurysms or arteriovenous fistulas [8]. Non-conservative treatments include minimally-invasive angiographic interventions or surgical revision, depending on hospital standards and available resources [7], [9]. Conservative treatments consist of close monitoring and blood transfusions when needed. However, allogenic blood transfusions in the perioperative setting are controversial. Transfusions are associated with higher perioperative mortality and morbidity and are challenged by limited availability. A potential immunosuppressive effect of transfusions and their possible impact on oncologic outcomes have been discussed [10], [11], [12]. Nevertheless, blood transfusion remains crucial in bleeding situations, and transfusion rates in RAPN range from 5–21% [13].

To our knowledge postoperative hemorrhage and associated factors for these events in RAPN have been analyzed in small, mostly monocentric datasets so far [14], [15], [16], [17]. To detect patients at risk in perioperative settings, this study focuses on identifying clinical factors associated with bleeding complications during RAPN in a multicenter dataset.

## 2 Patients and methods

This analysis includes RAPN performed consecutively by 41 different surgeons between June 2007 and June 2024 in nine robotic centers in Austria and Germany. Cases of RAPN were included consecutively without further selection. Surgery was performed with different robotic systems (DaVinci SI, X and XI, Intuitive Surgical, Inc., Sunnyvale, CA 94086-5304, United States of America), depending on accessibility in the hospitals. Surgical techniques (trans- vs retroperitoneal approach and clamping technique) were determined by surgeons’ preferences, as well as their expertise and tumor characteristics. Data were collected and analyzed retrospectively. Sporadic missing data of the original dataset were handled by a combination of mean imputation for age and body mass index (BMI) (*n* = 15) and exclusion of cases due to missing key variables (missing preoperative aspects and dimensions used for an anatomical [PADUA] score [*n* = 294] and missing blood values [*n* = 34]). Ninety-one percent (3726 cases) of the original dataset could be included in our analysis.

Demographic data were available for most of the patients. Tumor characteristics, including tumor complexity according to PADUA score and risk groups [18], and the perioperative course were obtained. Postoperative complications were evaluated according to the Clavien-Dindo classification [19]. To assess the impact of surgeons’ experience, the consecutive individual RAPN numbers of each surgeon (EXP) were considered. Continuous variables are described as the median and interquartile range (IQR), and categorical variables as absolute frequency and percentage.

Postoperative bleeding requiring intervention (PBRI) was defined as Clavien-Dindo grade 3 complication caused by bleeding requiring any surgical, radiological, or endoscopic intervention, and represented the primary endpoint of this study. Perioperative transfusion was defined as transfusion performed intra- and/or postoperatively, and was the secondary endpoint. We hypothesized that patient, tumor, and intraoperative characteristics are associated with the primary endpoint (PBRI) and the secondary endpoint (transfusion).

SPSS (version 29.0.2.0) was used for statistical analysis. Statistical significance was defined as *p* < 0.05. To screen clinically significant variables for the primary endpoint (PBRI), patients with PBRI were compared with those without PBRI. Continuous variables were analyzed using the Mann-Whitney U test, and categorical variables using the chi-squared test. For the secondary endpoint (transfusion), patients who received transfusion were compared to those without the need for transfusion using the same tests. All tests were two-sided. Subsequently, univariate logistic regression for clinically relevant variables was performed for each outcome separately. Variables showing statistical significance in univariate analyses were considered for inclusion in two separate multivariable logistic regression models, one for PBRI and one for transfusion. Model assumptions for multivariable logistic regression were assessed, including multicollinearity (variance inflation factors), independence of observations (Durbin-Watson statistic), and log-linearity of continuous variables (Box-Tidwell test), performed solely as an assumption check for model validity. To assess potential non-linear effects of the continuous variable age, we fitted a restricted cubic spline with four knots, and compared the model with a linear model using a likelihood ratio test (LRT) and the area under the receiver operating characteristic curve (AUC). The occurrence of PBRI and transfusion were defined as dependent variables in the respective regression analysis. Independent variables included EXP, patient’s age, BMI, American Society of Anesthesiologists (ASA) score, number of tumors, tumor size, operative and ischemia time, preoperative hemoglobin (Hb), glomerular filtration rate (GFR), surgical approach, and exophytic rate for regression analysis regarding transfusion. Independent variables included in regression analysis of PBRI were EXP, patient’s age, BMI, preoperative Hb, operative and ischemia time, and ASA score. Operative time was rescaled per 10 min to improve interpretability of odds ratios in the multivariable models. ASA score was grouped in ASA 1–2 versus ASA 3–4 due to small case numbers in ASA 4 for multivariate analysis. To investigate potential surgeon-specific or center-specific effects, the intraclass correlation coefficients (ICCs) for both dependent variables were calculated.

The study was approved by the ethics committee of Hannover Medical School (approval no. 11026_BO_K_2023), and all procedures performed in this study involving human participants were in accordance with the ethical standards of the institutional research committee and with the 1964 Helsinki Declaration and its later amendments. Informed consent was obtained. All participating institutions contributed data under existing data-sharing agreements.

## 3 Results

### 3.1 Patients’ characteristics

A total of 3726 patients were included in the analysis. Median age of the entire cohort was 64 yr (interquartile range [IQR] 56–72 yr), with a median BMI of 28 kg/m^2^ (IQR 25–31 kg/m^2^). Sixty-five percent of patients were male, and 59% had an ASA score of 2. Median preoperative Hb and estimated GFR (eGFR) were 14.4 g/dl (IQR 13.4–15.3 g/dl) and 76 ml/min/1.73 m^2^ (IQR 63–88 ml/min/1.73 m^2^), respectively. Median tumor size was 30 mm (IQR 20–42 mm) with a median PADUA score of 8 (IQR 7–10), and malignancy was observed in 74% of patients. Multiple tumors were present in 4.4.% of patients. Regarding surgical strategy, 87% of RAPN were performed transperitoneally, with an on-clamp technique used in 88% of patients, with a median ischemia time of 12 min (IQR 8–17 min) and a median operative time of 145 min (IQR 115–184 min). Median surgeon’s experience in RAPN was 72 cases (IQR 30–144 cases). Complications occurred in 17% of patients, with major complications (Clavien-Dindo > 2) in 7%. MIC-criteria (negative surgical margin, no major complication, and ischemia time under 20 min) were achieved in 90% of patients. Hemorrhage necessitating transfusion or PBRI occurred in 5.2% of patients.

### 3.2 Characteristics of patients with PBRI and predictors for PBRI in RAPN

PBRI occurred in 1.9% of patients. In 49% of PBRI cases, angiographic intervention was performed. Other interventions were operative revision (39%), nephrectomy (7.1%), endourologic interventions (7.1%), and drain placement (5.7%). Some patients necessitated multiple interventions. A higher rate of angiographic interventions was observed among all cases with PBRI in the recent period (2016–2024) compared with the period 2008–2015 (61% vs 41%; *p* > 0.05). Reasons for PBRI were arterial bleeding, arteriovenous fistula, or pseudoaneurysms.

Patients with PBRI were older (age 72 yr [IQR 59–77 yr] vs 64 yr [IQR 56–72 yr], *p* < 0.001), with higher ASA scores (ASA 3 in 39% vs 33%, ASA 4 in 7.8% vs 0.8 %, *p* < 0.001) and lower preoperative Hb (13.7 g/dl [IQR 12.7–15.0 g/dl] vs 14.4 g/dl [IQR 13.4–15.3 g/dl], *p* = 0.002). No differences were observed regarding patient’s gender, BMI, or preoperative renal function. The anatomic and histopathological tumor characteristics demonstrated no difference between patients with PBRI and those without PBRI. Median operative and ischemia time were longer in patients with PBRI (158 min [IQR 123–209 min] vs 145 min [IQR 115–183 min], *p* = 0.04 and 15 min [IQR 10–20 min] vs 12 min [IQR 8–17 min], *p* < 0.001, respectively), and surgeons performing RAPN with PBRI were less experienced in RAPN (52 cases [IQR 18–112 cases] vs 72 cases [IQR 30–145 cases], *p* = 0.02). Tumor resection were mostly performed with on-clamp technique (96% vs 88%, *p* > 0.05) and a transperitoneal approach (88% vs 87%, *p* > 0.05) (Table 1). Perioperative transfusion was required in 66% of patients. ICC for center and surgeons were both low (<0.1).

**Table 1:** Comparative analysis of RAPN with and without bleeding requiring intervention

Parameter	RAPN without bleeding requiring intervention(*n* = 3656)	RAPN with bleeding requiring intervention(*n* = 70)	*p* value *	Effect size **
Patients’ characteristics				
Age, yr (IQR)	64 (56–72)	72 (59–77)	**<0.001**	0.11
Male sex, *n* (%)	2383 (65)	49 (70)	0.4	0.01
BMI, kg/m^2^ (IQR)	27.7 (24.7–30.9)	27.5 (24.4–29.6)	0.3	0.03
CCI (IQR)	3 (1–6)	2 (1–6)	0.9	0.003
ASA score, *n* (%)				
1	235 (7.2)	1 (1.6)	**<0.001**	0.11
2	1918 (59)	33 (52)		
3	1079 (33)	25 (39)		
4	26 (0.8)	5 (7.8)		
Preoperative Hb, g/dl (IQR)	14.4 (13.4–15.3)	13.7 (12.7–15.0)	**0.002**	0.10
Preoperative eGFR, ml/min (IQR)	76 (63–89)	74 (59.1–87.7)	0.3	0.04
Tumor-specific characteristics				
Number of tumors, *n* (IQR)	1 (1–1)	1 (1–1)	0.2	0.04
Multiple tumors, *n* (%)	163 (4.5)	1 (1.4)	0.2	0.02
Tumor size, mm (IQR)	30 (20–42)	30 (24–35)	0.8	0.007
PADUA score (IQR)	8 (7–10)	8 (7–10)	0.2	0.04
Distribution of PADUA-risk groups				
Low (6–7), *n* (%)	1271 (35)	32 (46)	0.08	0.04
Intermediate (8–9), *n* (%)	1332 (36)	17 (24)		
High (> 10), *n* (%)	1053 (29)	21 (30)		
Intraoperative course				
Retroperitoneal approach, *n* (%)	466 (13)	8 (12)	0.7	0.006
Operative time, min (IQR)	145 (115–183)	158 (123–209)	**0.04**	0.07
Use of warm ischemia, *n* (%)	3153 (88)	66 (96)	0.053	0.03
Warm ischemia time, min (IQR)	12 (8–17)	15 (10–20)	**<0.001**	0.11
Surgeons’ experience in RAPN (EXP), *n* (IQR)	72 (30–145)	52 (18–112)	**0.02**	0.08
Postoperative course				
Postoperative complications, *n* (%)				
None	3102 (84)	0 (0.0)	**<0.001**	0.52
Clavien-Dindo 1	85 (2.3)	0 (0.0)		
Clavien-Dindo 2	279 (7.6)	0 (0.0)		
Clavien-Dindo 3	154 (4.2)	62 (88.6)		
Clavien-Dindo 4	32 (0.9)	8 (11.4)		
Clavien-Dindo 5	4 (0.1)	0 (0.0)		
Achievement of MIC criteria, *n* (%)	3297 (92.0)	0 (0.0)	**<0.001**	0.42
Histopathologic results				
Malignant histology, *n* (%)	2687 (74)	57 (81)	0.1	0.02
Tumor stage, *n* (%)				
pT1	2457 (93)	54 (96)	0.5	0.04
pT2	63 (2.4)	1 (1.8)		
pT3	122 (4.6)	1 (1.8)		
pT4	3 (0.1)	0 (0.0)		

ASA = American Society of Anesthesiologists; BMI = body mass index; CCI = Charlson comorbidity Index; eGFR = estimated glomerular filtration rate; Hb = hemoglobin; IQR = interquartile range; PADUA = preoperative aspects and dimensions used for an anatomical; RAPN = robot-assisted partial nephrectomy.

Continuous variables are shown as medians and IQR.

MIC criteria defined as negative surgical margin, no major complication, and ischemia time under 20 min.

Bold was used to indicate significant *p*-values.

* The *p* values were calculated for group comparisons using the Mann-Whitney U test (continuous variables) and the chi-squared test (categorical variables).

** Effect size is provided for continuous variables as Cohen's d and for categorical variables as Cramer-V.

Multivariable logistic regression analysis found preoperative Hb (odds ratio [OR] 0.77, 95% confidence interval [CI] 0.66–0.90, *p* = 0.001), ischemia time (OR 1.04, 95% CI 1.01–1.07, *p* = 0.01), and patient’s age (*p* = 0.009) as significant predictors of PBRI (Table 3a). No relevant violations of model assumptions were observed. The Box-Tidwell test to check log-linearity indicated a deviation from log-linearity for patient’s age. Age was therefore modeled using restricted cubic splines, which showed significant improvement of the model (LRT *p* = 0.03, AUC 0.70 vs 0.68).

### 3.3 Characteristics of patients requiring blood transfusion and predictors for blood transfusion in RAPN

Perioperative blood transfusion was necessary in 4.6% of the cohort. The majority of transfusions (84%) were administered postoperatively. Transfused patients were significantly older (71 yr [IQR 61–77 yr] vs 64 yr [IQR 56–72 yr], *p* < 0.001), with lower BMI (26.8 kg/m^2^ [IQR 24.2–30.2 kg/m^2^] vs 27.7 kg/m^2^ (IQR 24.7–30.9 kg/m^2^, *p* = 0.049), higher ASA scores (ASA 3 in 55% vs 32%, ASA 4 in 3.3% vs 0.8%, *p* < 0.001) and lower preoperative Hb and eGFR (13.2 g/dl [IQR 12.1–14.4 g/dl] vs 14.5 g/dl [IQR 13.5–15.3 g/dl] and 68 ml/min/1.73 m^2^ [IQR 52–80 ml/min/1.73 m^2^] vs 77 ml/min/1.73 m^2^ [IQR 63–89 ml/min/1.73 m^2^], respectively, both *p* < 0.001). Tumors in transfused patients were larger (33 mm [IQR 25–45 mm] vs 30 mm [IQR 20–42 mm], *p* = 0.005), and the percentage of patients with multiple tumors was higher (8.2% vs 4.2%, *p* = 0.01). No difference was found regarding PADUA score or histopathological characteristics (all *p* > 0.05). However, tumors were more often exophytic in the transfused group (exophytic part of the tumor > 50% in 61% vs 50% of cases, exophytic part of the tumor < 50% in 31% vs 38% of cases, and endophytic in 8.6% vs 11% of cases, *p* = 0.03), without difference in sinus or pelvic system infiltration. In both groups, resection was performed predominantly with on-clamp technique (91% vs 88%, *p* > 0.05) with longer ischemia and operative time in the transfused cohort (14 min [IQR 9–19 min] vs 12 min [IQR 8–17 min] and 168 min [IQR 132–228 min] vs 144 min [IQR 115–181 min], respectively, both *p* < 0.001). The retroperitoneal approach was used less frequently (7.7% vs 13%, *p* = 0.03) and surgeon’s expertise in RAPN was lower (53 cases [IQR 22–124 cases] vs 72 cases [IQR 31–145 cases], *p* = 0.005). Major complications occurred more often in the transfused group (Clavien-Dindo 3 in 35% vs 4.4%, Clavien-Dindo 4 in 8.8% vs 0.7 %, and Clavien-Dindo 5 in 0.6% vs 0.1%, *p* < 0.001) (Table 2). ICC for center and surgeons were both low (<0.1).

**Table 2:** Comparative analysis of RAPN with and without transfusion

Parameter	RAPN without blood transfusion (*n* = 3555)	RAPN with blood transfusion (*n* = 171)	*p* value*	Effect size**
Patients’ characteristics				
Age, yr (IQR)	64 (56–72)	71 (61–77)	**<0.001**	0.18
Male sex, *n* (%)	2330 (66)	102 (60)	0.1	0.03
BMI, kg/m^2^ (IQR)	27.7 (24.7–20.9)	26.8 (24.2–30.2)	**0.049**	0.07
CCI (IQR)	3 (1–5)	4 (1.5–9)	**<0.001**	0.15
ASA score, *n* (%)				
1	229 (7.2)	7 (4.7)	**<0.001**	0.12
2	1896 (60)	55 (37)		
3	1021 (32)	83 (55)		
4	26 (0.8)	5 (3.3)		
Preoperative Hb, g/dl (IQR)	14.5 (13.5–15.3)	13.2 (12.1–14.4)	**<0.001**	0.31
Preoperative eGFR, ml/min (IQR)	77 (63–89)	68 (52–80)	**<0.001**	0.17
Tumor-specific characteristics				
Number of tumors, *n* (IQR)	1 (1–1)	1 (1–20)	**0.02**	0.08
Multiple tumors, *n* (%)	150 (4.2)	14 (8.2)	**0.01**	0.04
Tumor size, mm (IQR)	30 (20–42)	33 (25–45)	**0.005**	0.09
PADUA score (IQR)	8 (7–10)	8 (7–10)	0.8	0.009
Distribution of PADUArisk groups				
Low (6–7), *n* (%)	1245 (35)	58 (33)	0.9	0.006
Intermediate (8–9), *n* (%)	1285 (36)	64 (37)		
High (> 10), *n* (%)	1025 (29)	49 (29)		
Intraoperative course				
Retroperitoneal approach, *n* (%)	461 (13)	13 (7.7)	**0.03**	0.04
Operative time, min (IQR)	144 (115–181)	168 (132–228)	**<0.001**	0.19
Use of warm ischemia, *n* (%)	3072 (88)	147 (91)	0.2	0.02
Warm ischemia time, min (IQR)	12 (8–17)	14 (9–19)	**<0.001**	0.12
Surgeons’ experience in RAPN (EXP), *n* (IQR)	72 (31–145)	53 (22–124)	**0.005**	0.09
Postoperative course				
Postoperative complications, *n* (%)				
None	3094 (87)	8 (4.7)	**<0.001**	0.51
Clavien-Dindo 1	85 (2.4)	0 (0.0)		
Clavien-Dindo 2	191 (5.4)	88 (52)		
Clavien-Dindo 3	157 (4.4)	59 (35)		
Clavien-Dindo 4	25 (0.7)	15 (8.8)		
Clavien-Dindo 5	3 (0.1)	1 (0.6)		
Achievement of MIC criteria, *n* (%)	3212 (92)	85 (53)	**<0.001**	0.23
Histopathologic results				
Malignant histology, *n* (%)	2607 (74)	137 (80)	0.053	0.03
Tumor stage, *n* (%)				
pT1	2391 (93)	120 (90)	0.4	0.04
pT2	61 (2.4)	3 (2.2)		
pT3	112 (4.4)	11 (8.2)		
pT4	3 (0.1)	0 (0.0)		

ASA = American Society of Anesthesiologists; BMI = body mass index; CCI = Charlson comorbidity Index; eGFR = estimated glomerular filtration rate; Hb = hemoglobin; IQR = interquartile range, PADUA = preoperative aspects and dimensions used for an anatomical; RAPN = robot-assisted partial nephrectomy.

Continuous variables are shown as medians and IQR.

MIC criteria defined as negative surgical margin, no major complication, ischemia time under 20 min.

* The *p* values were calculated for group comparisons using the Mann-Whitney U test (continuous variables) and the chi-squared test (categorical variables).

** Effect size is provided for continuous variables as Cohen's d and for categorical variables as Cramer-V.

**Table 3a:** Uni-/multivariable analysis for predictors of bleeding requiring intervention

	Univariate a			Multivariable (*n* = 3220)	
	OR (95% CI)	*p* value	*n*	OR (95% CI)	*p* value
Surgeon’s experience	1.00 (1.00–1.00)	0.08	3706		
Patient age b	1.04 (1.01–1.06)	**0.002**	3726	–	**0.009**
BMI	0.96 (0.92-1.01)	0.1	3725		
Preoperative Hb	0.73 (0.64–0.84)	**<0.001**	3726	0.77 (0.66–0.90)	**0.001**
Operative time c	1.05 (1.01–1.08)	**0.02**	3687	1.03 (1.00–1.08)	0.2
Ischemia time	1.05 (1.02–1.08)	**<0.001**	3650	1.04 (1.01–1.07)	**0.01**
ASA score 1–2	Ref.			Ref.	
ASA score 3–4	1.72 (1.05–2.82)	0.2	3222	1.24 (0.72–2.13)	0.4

ASA = American Society of Anesthesiologists; BMI = body mass index; CI = confidence interval; Hb = hemoglobin; IQR = interquartile range; OR = odds ratio.

Nagelkerkes R^2^ for multivariable analysis = 0.063

a Univariate analyses are presented for screening purposes only, interpretation in terms of content was not performed.

b Modelled as restricted cubic spline in multivariable logistic regression analysis, *p* value computed using simultaneous test for all splines terms.

c Operative time is scaled in 10-min slots.

Multivariable logistic regression analysis identified a significant association of higher patient age (OR 1.03, 95% CI 1.01–1.04, *p* = 0.01), ASA 3–4 (OR 1.92, 95% CI 1.29–2.88, *p* = 0.001), lower preoperative Hb (OR 0.61, 95% CI 0.54–0.68, *p* < 0.001), and longer operative time (OR 1.07, 95% CI 1.04–1.11, *p* < 0.001) with blood transfusion in RAPN (Table 3b). No relevant violations of model assumptions were observed. Box-Tidwell testing for log-linearity showed deviation for patient’s age; the effect estimate and 95% CI in the corresponding test statistics were close to 1 (OR 1.17, 95% CI 1.08–1.26). Non-linear modeling of age with restricted cubic splines did not improve the model significantly (LRT *p* = 0.1, AUC 0.80 vs 0.79); therefore, age was retained as linear covariable in this model.

**Table 3b:** Uni-/multivariable analysis for predictors of blood transfusion in RAPN

	Univariate a			Multivariable (*n* = 2885)	
	OR (95% CI)	*p* value	*n*	OR (95% CI)	*p* value
Surgeon’s experience	1.00 (1.00–1.00)	**0.007**	3706	1.00 (1.00–1.00)	0.5
Patient age b	1.04 (1.03–1.06)	**<0.001**	3726	1.03 (1.01–1.04)	**0.01**
BMI	0.98 (0.95–1.01)	0.2	3725		
Preoperative Hb	0.59 (0.54–-0.65)	**<0.001**	3726	0.61 (0.54–0.68)	**<0.001**
Preoperative eGFR	0.98 (0.97–1.00)	**<0.001**	3706	1.00 (0.99–1.01)	0.7
Number of tumors	1.45 (1.11–1.91)	**0.007**	3605	1.20 (0.95–1.53)	0.1
Tumor size	1.01 (1.00–1.02)	**0.04**	3723	1.00 (0.98–1.01)	0.5
Operative time c	1.09 (1.06–1.11)	**<0.001**	3687	1.07 (1.04–1.11)	**<0.001**
Ischemia time	1.04 (1.02–1.06)	**<0.001**	3650	1.01 (0.98–1.04)	0.5
ASA score 1-2	Ref.			Ref.	
ASA score 3-4		0.9	3322	1.92 (1.29–2.88)	0.001
Transperitoneal approach	Ref.			Ref.	
Retroperitoneal approach	0.54 (0.30–0.97)	**0.04**	3621	0.60 (0.30–1.17)	0.1
P_exophytic rate		**0.04**	3488		0.1
P_exophytic >50%	Ref.			Ref.	
P_exophytic <50%	0.67 (0.47–0.94)	**0.02**		0.68 (0.45–1.04)	0.08
P_endophytic	0.62 (0.35–1.10)	0.1		0.61 (0.30–1.25)	0.2

ASA = American Society of Anesthesiologists; BMI = body mass index; CI = confidence interval; eGFR = estimated glomerular filtration rate; Hb = hemoglobin; IQR = interquartile range; OR = odds ratio.

Nagelkerkes R^2^ for multivariable analysis = 0.185.

a Univariate analyses are presented for screening purposes only, interpretation in terms of content was not performed.

b Modelled as linear covariable.

c Operative time is scaled in 10-min slots.

## 4 Discussion

Postoperative hemorrhage is the main complication after partial nephrectomy and represents a potentially life-threatening event [5]. In our cohort, hemorrhage necessitating transfusion or PBRI occurred in 5.2%, including PBRI in 1.9% and blood transfusions in 4.6%. These findings are in accordance with prior studies, confirming that such complications are rare events and supporting the safety of nephron-sparing surgery with RAPN [5], [7], [13]. Hemorrhagic complications in partial nephrectomy have been analyzed previously [15], [16], [17], [20]. However, most prior studies were based on unicentric datasets with fewer than 1000 cases and summarized both minimally invasive and open surgical approaches or generalized bleeding complications.

In our cohort, the primary management strategy for PBRI was angiographic intervention, a minimally invasive, well-established technique that allows the preservation of the operated kidney. This procedure is characterized by femoral artery catheterization, by identification of the bleeding source through arteriography, and by selective embolization of the vascular lesion without impairing the blood supply to the remaining kidney [21]. Another frequent therapeutic strategy was surgical revision, with nephrectomy required in 7.1% of cases. Therefore, the operated kidney could be preserved in the majority of hemorrhagic complications. However, kidney-preserving strategies demand the availability of interventional radiology expertise or skilled open surgeons. The relevance of surgeon’s expertise in open surgery on kidney preservation in RAPN complications has been demonstrated previously in a study by our working group. Surgeons with greater open surgical experience were more likely to convert to open partial rather than radical nephrectomy during RAPN complications [22]. This analysis further demonstrated that surgeon’s robotic experience in renal surgery was lower in RAPN with either transfusion or PBRI, although multivariable analysis showed no significant association. Learning-curve analysis in RAPN recently found an association between fewer major complications and increasing expertise in RAPN [23]. However, this effect was not observed in other studies [24], [25].

Patient’s characteristics were significantly associated with both transfusion requirements and PBRI in RAPN. In multivariable analysis, lower preoperative Hb-levels and patient’s age were predictive of both complications, while ASA score 3–4 was only associated with transfusion. The association between preoperative anemia and postoperative transfusion is plausible given the reduced baseline Hb levels. Previous studies have linked preoperative anemia to a higher risk of overall postoperative complications in partial nephrectomy, though a specific association with hemorrhage has not been described yet [26]. In colorectal surgery, severe preoperative anemia has been identified as a predictor for postoperative non-hemorrhagic complications and blood transfusions, and preoperative iron supplementation could reduce both transfusion rates and complications in general [27]. A randomized controlled study investigating preoperative iron supplementation in patients with iron deficiency before abdominal surgery showed a reduction of transfusion rates and hospital stay in the supplementation group [28]. However, iron supplementation prior to surgery is currently not an established practice in urologic surgery. Although our results showed a statistical association, a causal interpretation is not possible. Nevertheless, our results raise the question of whether patients with anemia might benefit from prehabilitation programs, including anemia diagnostics and correction, as part of the preoperative preparation to partial nephrectomy. The median preoperative Hb in both complication groups was approximately 13 g/dl, bordering on the threshold for anemia. Further studies focusing on RAPN patients with anemia are required, especially to define a critical Hb-threshold to identify patients at increased risk.

Regarding ASA score and patient’s age, prior analysis identified an association with complications in partial nephrectomy; however, no association with hemorrhage specifically was found [17], [26]. Patients with higher ASA scores showed a statistically significant association only with transfusion. For patient’s age, the log-linearity assumption of the logistic regression model for transfusion was not fully met, and the effect should be interpreted with caution. However, the observed OR and 95% CI in the Box-Tidwell test to check log-linearity were close to 1, suggesting minimal clinical nonlinearity for patient’s age. Similarly, the ORs and 95% CIs for patient’s age in the multivariable logistic regression were also close to 1, indicating that the clinical effect of age is likely minimal. Additionally intraoperative characteristics, including operative time for transfusion risk and ischemia time for PBRI, were identified as predictors. This is in line with former findings associating longer operative and ischemia times with hemorrhagic complications in partial nephrectomy [15], [16]. Prolonged operative time may be related to a multitude of reasons ranging from challenging anatomic features like the presence of adhesive perinephric fat to a less experienced surgeon [23], [29]. In contrast to our results, Jung et al found no association between ischemia time and severe hemorrhage in partial nephrectomy [17]. Extended ischemia time may reflect a more complex tumor resection due to complex tumor anatomy. Notably, tumor-related characteristics (represented by the PADUA score), tumor size, multiple tumors, and histopathologic characteristics, showed no significant association with hemorrhagic complications in our cohort (*p* > 0.05). Previous studies report controversial results on this topic. Although some investigations did not report any association between tumor characteristics (tumor size, stage, and R.E.N.A.L score) and severe hemorrhage in partial nephrectomy, others found nephrometry scores predictive of bleeding in univariate but not in multivariable analyses [4], [15], [16], [17]. Furthermore, Saoud et al found higher R.E.N.A.L scores to be independently associated with hemorrhage requiring angioembolization [8]. Variability of results may be linked to different study designs. Some analysis summarized all surgical approaches [8], [16], [17], while others focused on an open or a robotic surgery [4], [15]. Our study focused exclusively on RAPN cases, questioning the role of surgical approach as potentially relevant co-factor in hemorrhage risk assessment.

Due to inclusion of multiple robotic centers and surgeons, we addressed potential center- or surgeon-specific effects via calculation of the ICC. ICC values were low; thus, clustering was minimal and multilevel logistic regression was not required.

Our study has several limitations. First, as a retrospective analysis, it is subject to lack of documentation and missing data. Some further relevant variables, such as preoperative anticoagulant use, prior ipsilateral renal surgery, the accurate intraoperative blood loss, clamping technique, or details on renorrhaphy techniques were not available. Despite the large sample size, residual confounding due to unmeasured factors cannot be entirely excluded, and missing data and exclusion of patients with incomplete information may have introduced selection bias, which could affect generalizability of our findings. Surgeries were performed across nine different centers by 41 different surgeons over a 17-yr period. No adjustment was made for center-specific evolutions or temporal changes in surgical techniques or technology. Furthermore, long-term follow-up was not available. Due to multiple univariable comparisons, there is a potential risk of type I error inflation. Therefore, findings from variable screening should be interpreted with caution. Although variables were primarily selected based on clinical relevance and plausibility, univariate analyses were additionally used to guide inclusion in the multivariable models. This approach may introduce bias in coefficient estimates. However, the risk of overfitting is low given the large sample size. Nevertheless, caution is warranted when generalizing the findings beyond the study population. Finally, our analysis identifies significant associations between patient and intraoperative characteristics and both PBRI and transfusion; nevertheless, we did not provide a clinical prediction tool. Further, randomized, prospective studies are required to evaluate whether risk-adapted clinical management based on the identified factors improve patient outcome.

## 5 Conclusions

Relevant hemorrhagic complications in RAPN are rare, and established interdisciplinary management strategies are crucial for kidney preservation. The main associated variables include patient-related and intraoperative characteristics. Particularly, patients with anemia and multimorbidity should be monitored intensely postoperatively, and blood product reservation should be considered. Future studies are required to assess whether preoperative optimization, such as anemia diagnostics and correction, is associated with reduced rates of PBRI or transfusions in RAPN.

  ***Author contributions:*** Olga Katzendorn had full access to all the data in the study and takes responsibility for the integrity of the data and the accuracy of the data analysis.

  *Study concept and design*: Katzendorn, Harke.

*Acquisition of data*: Katzendorn, Idais, Kanaan, Tabrizi, Schiefelbein, Schoen, Wiesinger, Gloger, Eraky, Ayanle, Kesch, Al-Nader, Münden, Fuhrmann, Zeuschner, Mayr, Harke.

*Analysis and interpretation of data*: Katzendorn, Kanaan, Zeuschner, Harke.

*Drafting of the manuscript*: Katzendorn.

*Critical revision of the manuscript for important intellectual content*: Katzendorn, Idais, Kanaan, Tabrizi, Schiefelbein, Schoen, Wiesinger, Ubrig, Gloger, Nuhn, Eraky, Wagner, Ayanle, Kesch, Al-Nader, Hadaschik, Münden, Fuhrmann, Kuczyk, Siemer, Zeuschner, Mayr, Harke.

*Statistical analysis*: Katzendorn, Kanaan, Zeuschner, Harke.

*Obtaining funding*: None.

*Administrative, technical, or material support*: Katzendorn, Zeuschner, Harke.

*Supervision*: Schiefelbein, Schoen, Ubrig, Nuhn, Wagner, Hadaschik, Kuczyk, Siemer, Harke.

*Other* (specify): None.

  ***Financial disclosures:*** Olga Katzendorn certifies that all conflicts of interest, including specific financial interests and relationships and affiliations relevant to the subject matter or materials discussed in the manuscript (eg, employment/affiliation, grants or funding, consultancies, honoraria, stock ownership or options, expert testimony, royalties, or patents filed, received, or pending), are the following: None.

  ***Funding/Support and role of the sponsor*:** None.
